# Uncovering the mechanism of resveratrol in the treatment of asthma: a network pharmacology approach with molecular docking and experimental validation

**DOI:** 10.3389/fphar.2025.1596737

**Published:** 2025-05-16

**Authors:** Li Chunxiao, Hou Xin, Liu Bowen, Sun Bingqing, Zhou Mengqi, Zhang Shuwen, Zhang Jingyuan, Lin Jiangtao

**Affiliations:** ^1^ Peking University China-Japan Friendship School of Clinical Medicine, Beijing, China; ^2^ Graduate School, Beijing University of Chinese Medicine, Beijing, China; ^3^ Graduate School of Peking Union Medical College, Chinese Academy of Medical Sciences/Peking Union Medical College, Beijing, China; ^4^ Department of Pulmonary and Critical Care Medicine, National Center for Respiratory Medicine, Peking University China-Japan Friendship School of Clinical Medicine, Beijing, China

**Keywords:** asthma, resveratrol, network pharmacology, molecular docking, airway inflammation

## Abstract

**Background:**

Evidence for the benefits of resveratrol (Res) in the treatment of asthma is progressively accumulating. However, the full spectrum of its molecular targets and the precise mechanisms remain incompletely characterized.

**Method:**

Targets of Res were obtained from Swiss Target Prediction, TCMCP, and DrugBank. Targets of asthma were obtained from DisGeNET, Therapeutic Target Database, GeneCards, and DrugBank. Intersecting target genes were identified by using jvenn. Gene Ontology (GO) and Kyoto Encyclopedia of Genes and Genomics (KEGG) enrichment analyses were performed using the R package clusterProfiler in R version 4.4.0. Protein–protein interaction networks were constructed using Cytoscape 3.9.1 software. Molecular docking validation of the binding capacity between Res and targets was performed using AutoDock Vina and visualized in PyMOL version 3.0.4. ELISA and Western blotting were used to verify the reliability of Res effects on the top five targets in both house dust mite (HDM)-induced asthma mouse model and BEAS-2B cell model.

**Results:**

After the intersection of the 236 Res targets and the 2,382 asthma targets, 120 targets for Res against asthma were obtained. The top five therapeutic targets based on weighted degree score were TNF, IL6, STAT3, TP53, and IL1B. GO enrichment analysis identified 2,595 significant terms, associated with 2,402 biological processes, followed by 153 molecular functions and 40 cellular components. KEGG enrichment analysis identified 107 relevant pathways, including “apoptosis,” “TNF signaling pathway,” and “MAPK signaling pathway.” Molecular docking showed that Res had a strong binding affinity toward the top five targets with binding energies less than −5.8 kcal/mol. Res treatment normalized the dysregulated expression of TNF-α, IL-6, STAT3, p53, and IL-1β both *in vitro* and *in vivo*.

**Conclusion:**

Res may target TNF-α, IL-6, STAT3, p53, and IL-1β to act as a therapeutic agent for asthma. These findings reveal the potential therapeutic targets for Res against asthma and provide theoretical bases for the clinical application of Res.

## 1 Introduction

Asthma, a heterogeneous disease impacting over 300 million individuals globally, results in approximately 250,000 deaths each year (Dharmage et al., 2019). In China, adults aged 14 years and above have a 1.24% prevalence rate of asthma (Lin et al., 2018). The adverse effects of prolonged oral corticosteroids (OCS) use and the high cost of biologics treatment place a heavy burden on both patients and the society.

Recent studies have revealed that various phytochemicals exhibit significant pharmacological activities in preclinical research (Nainwal et al., 2020; Arora et al., 2021; Arora et al., 2022a; Nainwal et al., 2022; Rahman et al., 2022; Jasemi et al., 2024; Nabi et al., 2024). Resveratrol (Res), a non-flavonoid polyphenol commonly found in plants such as grapes, peanuts, and berries, is primarily present in its trans form, known for its superior physiological activity compared to the cis form (Trela and Waterhouse, 1996; Wicinski et al., 2017). Res is metabolized primarily in the gastrointestinal tract and liver and is characterized by easy absorption, metabolism, and excretion (Wang and Sang, 2018). It is less distributed in lung tissue, but *in vivo* experiments have also demonstrated significant effects on the lung (Svilar et al., 2019). The multifaceted benefits of Res include anti-inflammatory, antioxidant, lipid-lowering, antiaging, and tumor-preventive properties (Csaki et al., 2008; Eo et al., 2013; Bellaver et al., 2014; Malaguarnera, 2019). It has been demonstrated that Res exerts therapeutic effects on a wide range of diseases, encompassing diabetes, cardiovascular disease, and cancer (Cai et al., 2020; Ren et al., 2021; Liu et al., 2022; Su et al., 2022; Rahimifard et al., 2023). A plethora of clinical trials have validated the therapeutic efficacy and low toxicity of Res (Brown et al., 2010; Howells et al., 2011; Beijers et al., 2020), and its clinical application is promising. Res has also exhibited beneficial effects in various asthma models (Lee et al., 2009; Chen et al., 2015; André et al., 2016; Li et al., 2018; Jiang et al., 2019). It can target different cell types involved in asthma pathophysiology, including airway epithelial cells (AECs), mast cells, and eosinophils (Han et al., 2015; Hu et al., 2016; Xu et al., 2020; Zeng et al., 2022). Nevertheless, the details of the targets and related pathways of Res in asthma remain to be elucidated.

Network pharmacology uses a combination of computational and experimental methods to integrate large amounts of information, thereby facilitating the identification of novel discoveries (Zhao et al., 2023; Muhammed and Aki-Yalcin, 2024). It has been demonstrated to be a potent instrument in elucidating the mechanisms of action of complex drug systems, with the potential to expedite the drug development process. Molecular docking elucidates intermolecular interactions by predicting three-dimensional (3D) binding configurations to guide rational drug design (Nainwal et al., 2014; Pinzi and Rastelli, 2019; Muhammed and Aki-Yalcin, 2024). Although Res has shown potential in asthma treatment, comprehensive network pharmacology studies in this area are still lacking.

**FIGURE 1:**
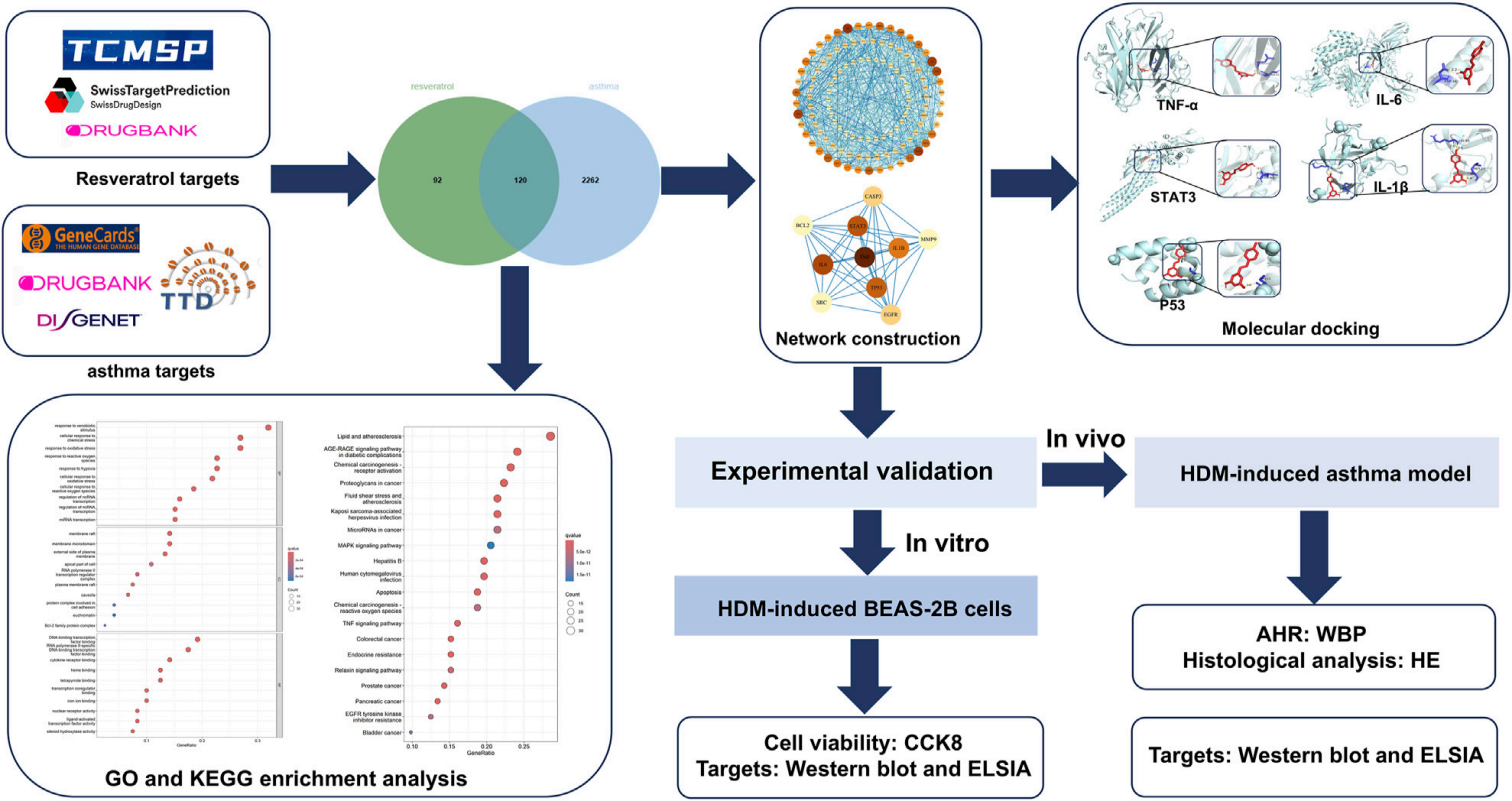
Research flowchart.

This study employed network pharmacology and molecular docking to identify Res-related targets and signaling pathways relevant to asthma intervention. To authenticate the credibility of the computational findings, we validated the effect of Res on target genes in both a house dust mite (HDM)-induced asthma mouse model and a BEAS-2B cell model. Given the results of this study, Res could potentially offer a new avenue for asthma management. The detailed framework of this investigation is visually depicted in Figure 1.

## 2 Materials and methods

### 2.1 Drug targets of RES

The chemical structure of Res was obtained, and the canonical SMILES were recorded through the PubChem database (https://pubchem.ncbi.nlm.nih.gov/, accessed in January 2025). Three databases, TCMCP (https://test.tcmsp-e.com/tcmsp.php, accessed in January 2025), Swiss Target Prediction (http://swisstargetprediction.ch/, accessed in January 2025), and DrugBank (https://go.drugbank.com/, accessed in January 2025), were combined to acquire target genes for Res. The data obtained from the aforementioned databases were merged, with any duplicates being removed.

### 2.2 Therapeutic targets for asthma

Four databases, DisGeNET (https://www.disgenet.org/, accessed in January 2025), Therapeutic Target Database (https://db.idrblab.net/ttd/, accessed in January 2025), GeneCards (https://www.genecards.org/, accessed in January 2025), and DrugBank (https://go.drugbank.com/, accessed in January 2025), were used to screen potential asthma targets. The merged and de-duplicated targets from the DisGeNET (GDAs score>0.06), GeneCards (relevance score>1), Therapeutic Target database, and DrugBank were selected as targets of asthma.

### 2.3 Protein–protein interaction networks

Intersections of Res and asthma targets were performed by jvenn (https://xcmsonline.scripps.edu/lib/jvenn/example.html). The STRING database (https://cn.string-db.org//, accessed in January 2025) was used to construct the PPI network, which was visualized in Cytoscape version 3.91 using the tsv file. The degree of interaction for each protein node within the constructed network was evaluated.

### 2.4 GO and KEGG enrichment analysis

Gene Ontology (GO) and Kyoto Encyclopedia of Genes and Genomics (KEGG) pathway analyses were completed using the R package clusterProfiler in R version 4.40. GO analyses comprise three main categories: molecular function (MF), which describes the biochemical activities of gene products; cellular component (CC), which defines their subcellular localization; and biological process (BP), which outlines broader physiological pathways. The top 10 GO annotations and top 20 KEGG pathways (*p* < 0.05) were assigned for further visualization.

### 2.5 Molecular docking

The top five hub genes were utilized for additional molecular docking to investigate the interaction of Res with targets. The 3D structure of Res was optimized using the MM2 molecular mechanics method in the Chem3D software. Hub protein structures were acquired from the RCSB Protein Data Bank (PDB, https://www.rcsb.org, accessed in January 2025). Water molecules and small ligands were deleted using PyMOL version 3.0.4, and hydrogen atoms were subsequently added with AutoDock Tools version 1.5.7. A grid box was generated around the active site of the receptor to facilitate subsequent molecular docking. Semi-flexible docking of the receptor to the corresponding ligand was performed using AutoDock Vina. The most stable receptor–ligand complexes were selected by evaluating the global minimum binding energy, hydrogen bonding interactions, and ligand occupancy within the active pocket. The optimal docking conformation was exported, followed by visualization of interacting residues and hydrogen bonds using PyMOL (Seeliger and de Groot, 2010; Parida et al., 2013; Nainwal et al., 2018). Two-dimensional (2D) visualization of molecular docking interactions was performed using LigPlot+ software.

### 2.6 The measurement of cell viability

BEAS-2B cells (Meisen, Cat# CTCC-400-0007) were treated with the Cell Counting Kit-8 reagent (Biosharp, Cat# BS350A) for the assessment of cell viability. Cells were plated in 96-well culture plates at a density of approximately 2 × 10^3^ cells per well. Cells were cultured and treated with Res at six different concentrations (0, 10, 20, 40, 80, and 100 μM) and stimulated 1 h later with 800 μM HDM (Biolead, Cat# XPB82D3A25). After treatment periods of 6, 12, 24, and 48 h, 10 μL CCK-8 was added to each well and incubated for 1–4 h. Absorbance was assessed at 450 nm.

### 2.7 Cell culture

DMEM/F12 medium (Gibco, Cat# 6124491) was used for cell culture. At 80% confluency, the cells were randomly distributed into the control, model, and Res groups. Cells were starved for 12 h after cell density reached approximately 70%, followed by 10 μM Res (MedChemExpress, Cat# HY-16561) treatment for 1 h. Following a 24-h stimulation period with 800 μM HDM, the cells were collected for subsequent research.

### 2.8 Animal and experiment design

An asthma model was established using female BALB/c mice (18–22 g, 6–8 weeks old, Beijing Vital River Laboratory Animal Technology Co., Ltd.). All relevant animal interventions were performed according to the ARRIVE guidelines. Mice were randomly assigned to three distinct groups (n = 7): (1) control group, (2) model group, and (3) Res group. The asthma model was constructed by HDM induction, and the experimental protocol was optimized according to the established sensitization paradigms (Johnson et al., 2004; Chen et al., 2018b; Yang et al., 2019; Daubeuf and Frossard, 2021). Sensitization phase: on days 0, 3, and 7, mice in the model and Res groups received intraperitoneal injections of 50 μg HDM (dissolved in 100 μL PBS), whereas mice in the control group received PBS alone. Challenge phase: from day 15 to day 21, mice in the model and Res groups were subjected to daily intranasal instillation of 50 μg HDM dissolved in 50 μL PBS. Prior to each HDM stimulation, resveratrol (100 mg/kg) was intraperitoneally injected daily into mice in the Res group, whereas mice in the control group received PBS following the same schedule. Airway hyperresponsiveness (AHR) was calculated, and the mice were euthanized on day 23.

### 2.9 Evaluations of AHR

Lung resistance was measured with the FinePointe Whole Body Volume Profiling System—WBP for assessment of AHR (Lim et al., 2014; Chen et al., 2018a; Varshneya et al., 2022). Following calibration and zeroing of the instrument, methacholine was nebulized at six levels (0, 3.125, 6.25, 12.5, 25, and 50 mg/mL). One lung function test time comprises several distinct stages: adaptation time of 10 min, nebulization time of 3 min, reaction time of 5 min, and recovery time of 1 min. As enhanced pause (Penh) demonstrates the highest sensitivity for reflecting AHR in murine models (Hamelmann et al., 1997; Flanagan et al., 2019; Yao et al., 2019; Hajimohammadi et al., 2020; Li et al., 2020), we quantitatively assessed AHR by measuring Penh values in this study.

### 2.10 Assessment of lung tissue inflammation

Lung tissue was fixed in 4% paraformaldehyde for ≥24 h. Paraffin embedding and sectioning were then performed. Tissue sections underwent hematoxylin and eosin (H&E) staining for histological evaluation, followed by microscopic examination and image acquisition. The lung tissue as a whole was observed at 50 magnifications, and the differences in alveoli, vessels, and trachea area were observed separately at 400 magnifications.

### 2.11 Western blotting

As intracellular proteins, the protein expression of p53 and STAT3 in lung tissue and AECs was assessed by Western blotting. Protein samples (50 μg per lane) were electrophoresed on a 10% SDS-PAGE gel and subsequently transferred onto PVDF membranes (0.45 μm; Millipore, Cat# IPVH00010) *via* wet blotting. Membranes were blocked for 10 min at room temperature using a protein-free rapid blocking buffer (Epizyme, Cat# PS108P), followed by overnight incubation with primary antibodies at 4°C under gentle agitation (β-actin: Proteintech, Cat# 66009-1-Ig; STAT3: Proteintech, Cat# 80149-1-RR; p53: Proteintech, Cat# 21891-1-AP). After washing, the membranes were then incubated with an HRP-conjugated goat anti-rabbit secondary antibody (Epizyme, Cat# LF102) for 1 h. ImageJ software was used to analyze protein bands, and the expression levels were normalized against β-actin.

### 2.12 Enzyme-linked immunosorbent assay

Secreted proteins, cell supernatants, and lung tissue homogenates were analyzed for IL-1β, IL-6, and TNF-α levels using ELISA. All ELISA kits were purchased from MultiSciences Biotech Co., Ltd. (Hangzhou, China). The experimental steps were performed according to the instructions, with four sub-holes per group. Finally, the OD values at 450 nm and 630 nm (or 570 nm) were measured. The calibration OD value is the measurement at 450 nm minus the measurement at 630 nm (or 570 nm).

### 2.13 Statistical analysis

GraphPad Prism 9.0 was employed for statistical analyses. A t-test was utilized to analyze differences between two groups, whereas one-way ANOVA was applied for comparisons across multiple groups. Data were expressed as mean ± standard deviation (SD), and *p* < 0.05 was deemed statistically significant.

## 3 Results

### 3.1 Acquisition of Res targets and asthma targets

**FIGURE 2:**
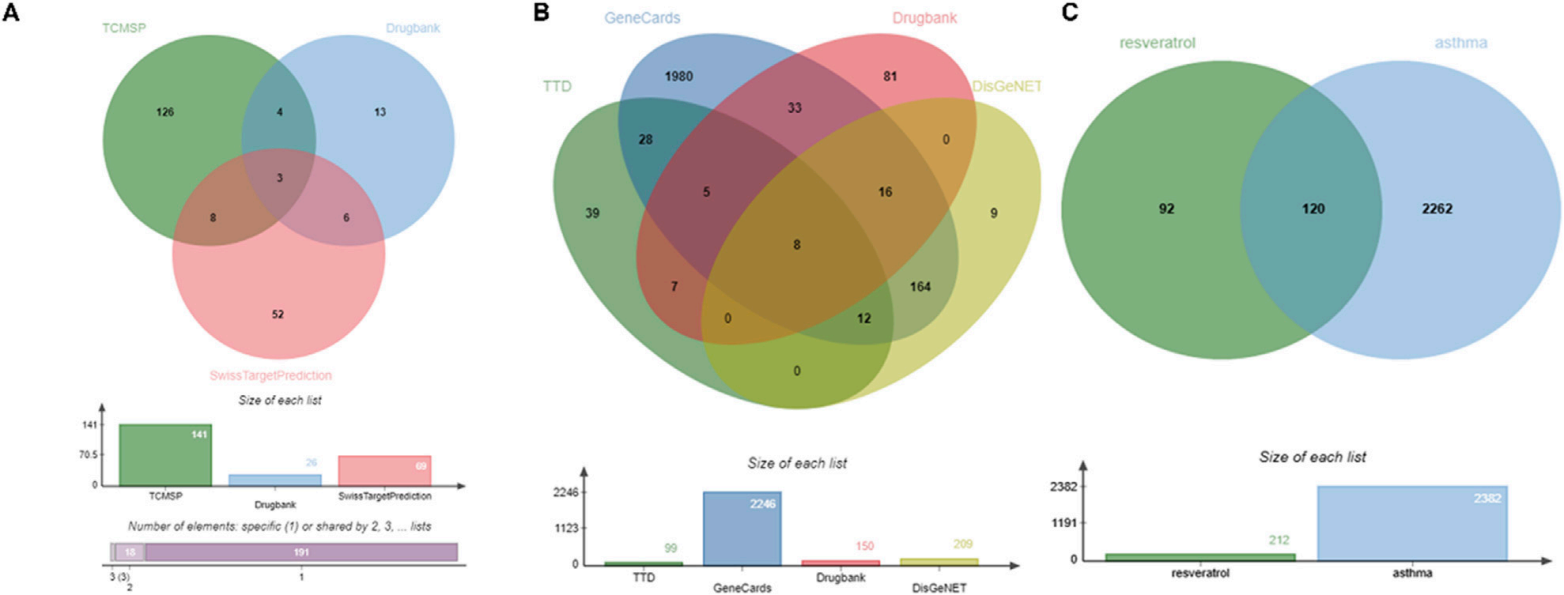
Identification of therapeutic targets for Res against asthma using Venn diagram. **(A)** Targets of Res. **(B)** Targets of asthma. **(C)** Intersection of Res and asthma genes.

The TCMCP, DrugBank, and SwissTargetPrediction databases contain 141, 26, and 69 Res targets, respectively. We combined the three database targets and removed 22 duplicates to obtain 236 Res targets for further analysis (Figure 2A). In the DrugBank, DisGeNET, GeneCards, and TTD databases, 150, 209, 2,246, and 99 asthma-related targets were identified, respectively. A total of 2,382 disease targets were obtained after removing 323 redundant targets (Figure 2B).

### 3.2 Acquisition of intersecting genes and construction of the PPI network

**FIGURE 3:**
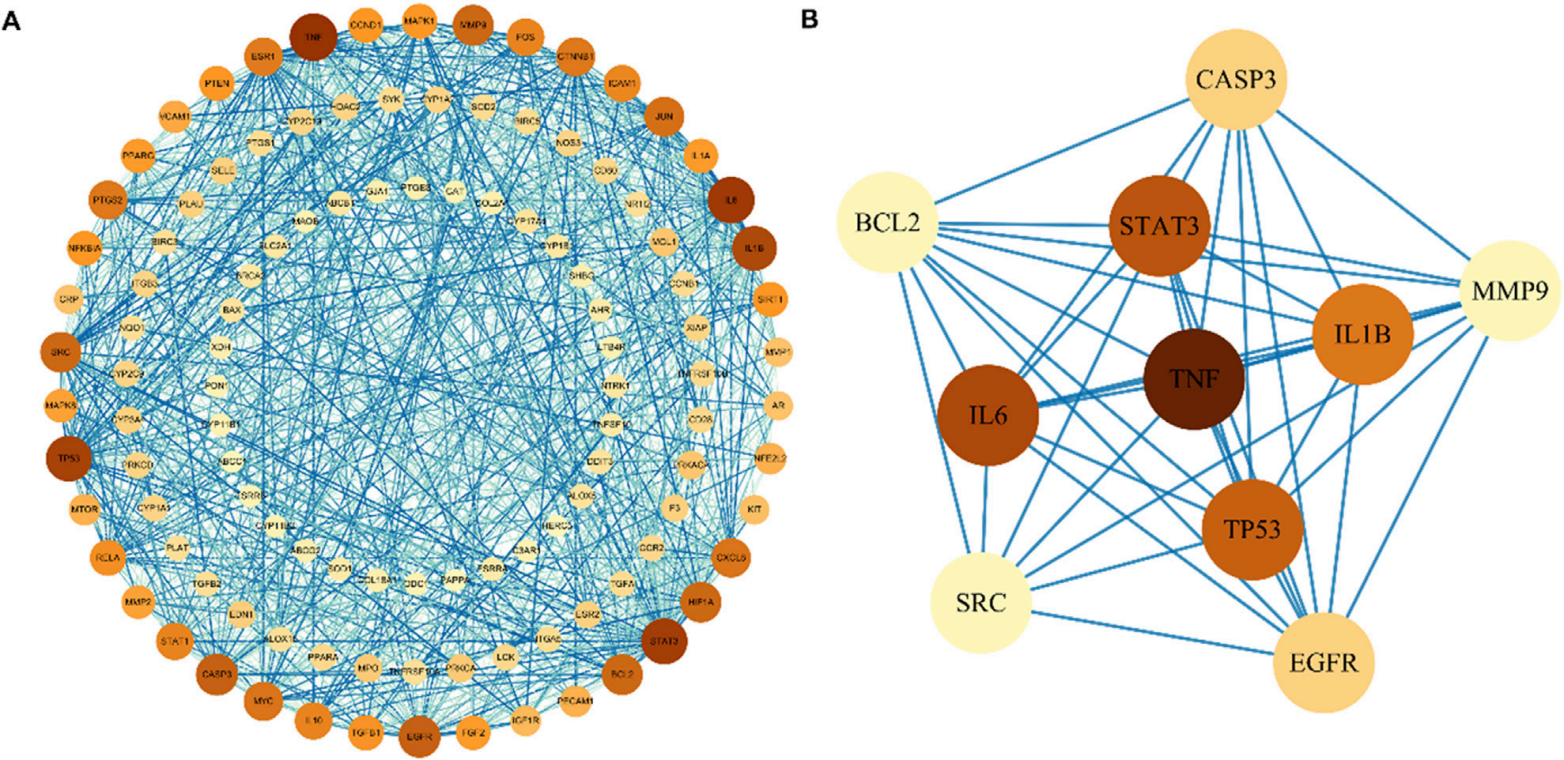
Protein–protein interaction network of Res targets against asthma. **(A)** Protein interactions of all intersecting genes. The color of the nodes reflects the degree of connectivity. **(B)** Protein interactions of the TOP 10 overlapping genes.

By intersecting the above Res targets with asthma targets, 120 potential Res targets for asthma were obtained (Figure 2C). After importing 120 targets into the STRING database, 113 nodes and 892 edges were obtained in the PPI network. The degree of the node is directly proportional to its size and darkness (Figure 3A). The top five therapeutic targets according to the weighted degree score were TNF, IL6, STAT3, TP53, and IL1B (Figure 3B).

### 3.3 GO and KEGG enrichment analyses

**FIGURE 4:**
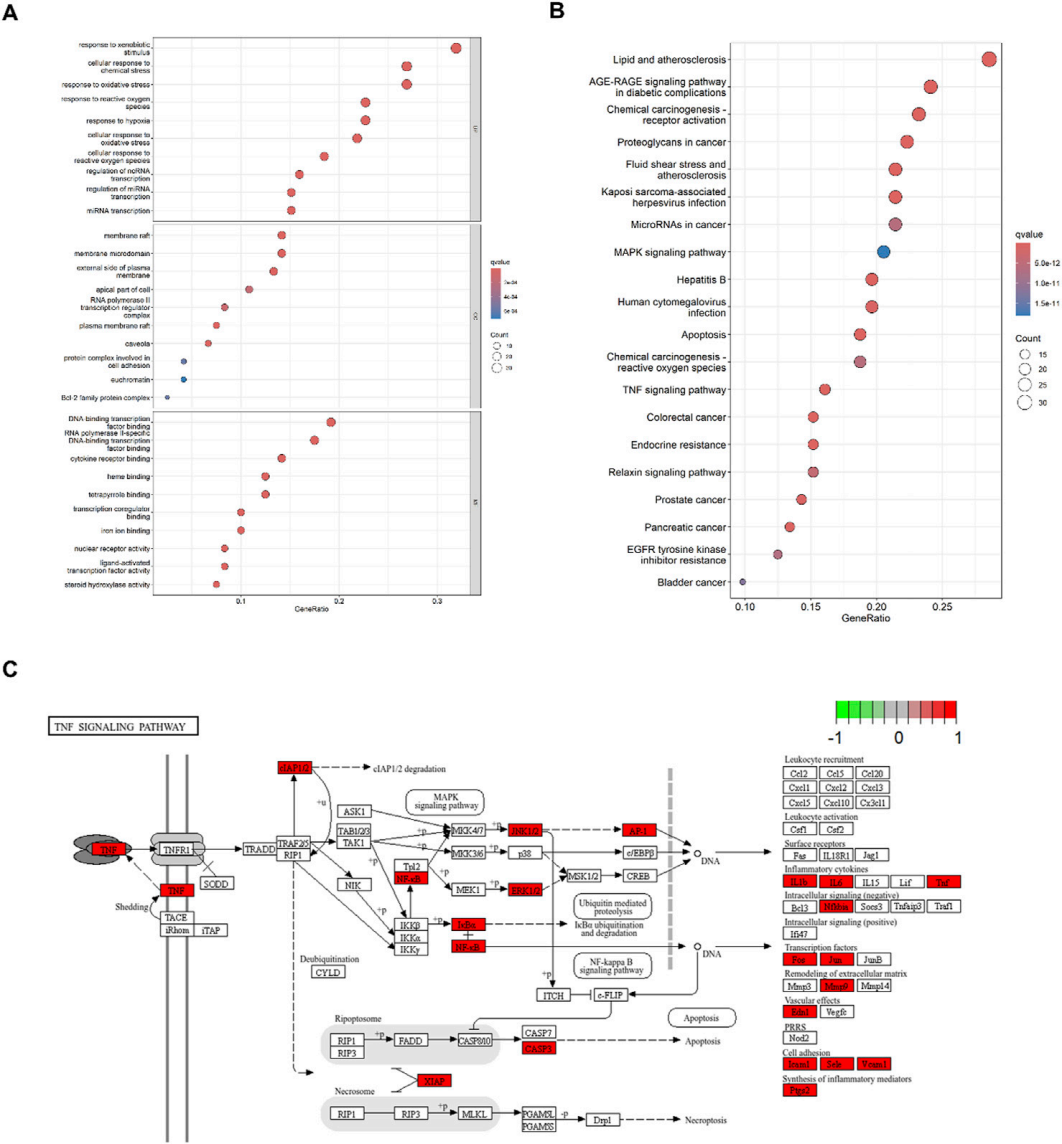
Enrichment analysis of 120 intersected targets. **(A)** Top 10 GO enrichment terms. BP: biological process, CC: cellular component, and MF: molecular function. **(B)** Top 20 KEGG enrichment analysis item. **(C)** TNF signaling pathway.

GO analysis identified 2,595 significant terms, which were predominantly associated with biological processes (2,402 terms, 92.6%), molecular functions (153 terms, 5.9%), and cellular components (40 terms, 1.5%). The top 10 CC, BP, and MF terms are shown in Figure 4A. The top enriched BP terms included “response to xenobiotic stimulus,” “cell response to chemical stress,” “response to oxidative stress,” and “response to hypoxia,” suggesting a potent modulatory effect on oxidative stress. For MF, these genes were associated with DNA-binding transcription factor binding, RNA polymerase II-specific DNA-binding transcription factor binding, and cytokine receptor binding, among others. In terms of CC, these genes participated in the membrane raft, membrane microdomain, external side of the plasma membrane, and apical part of the cell, among others. KEGG enrichment analysis identified 107 relevant pathways. The top 20 pathways in the list of results are shown in Figure 4B. The top enriched pathways include “apoptosis,” “TNF signaling pathway,” and “MAPK signaling pathway.” It indicates that Res may be involved in regulating inflammation and apoptosis, demonstrating its multi-targeted regulatory capacity. Notably, top three targets (TNF, IL1B, and IL6) were enriched in the TNF signaling pathway (Figure 4C), suggesting their potential coordination in Res’s anti-inflammatory action.

### 3.4 Molecular docking between Res and targets

**FIGURE 5:**
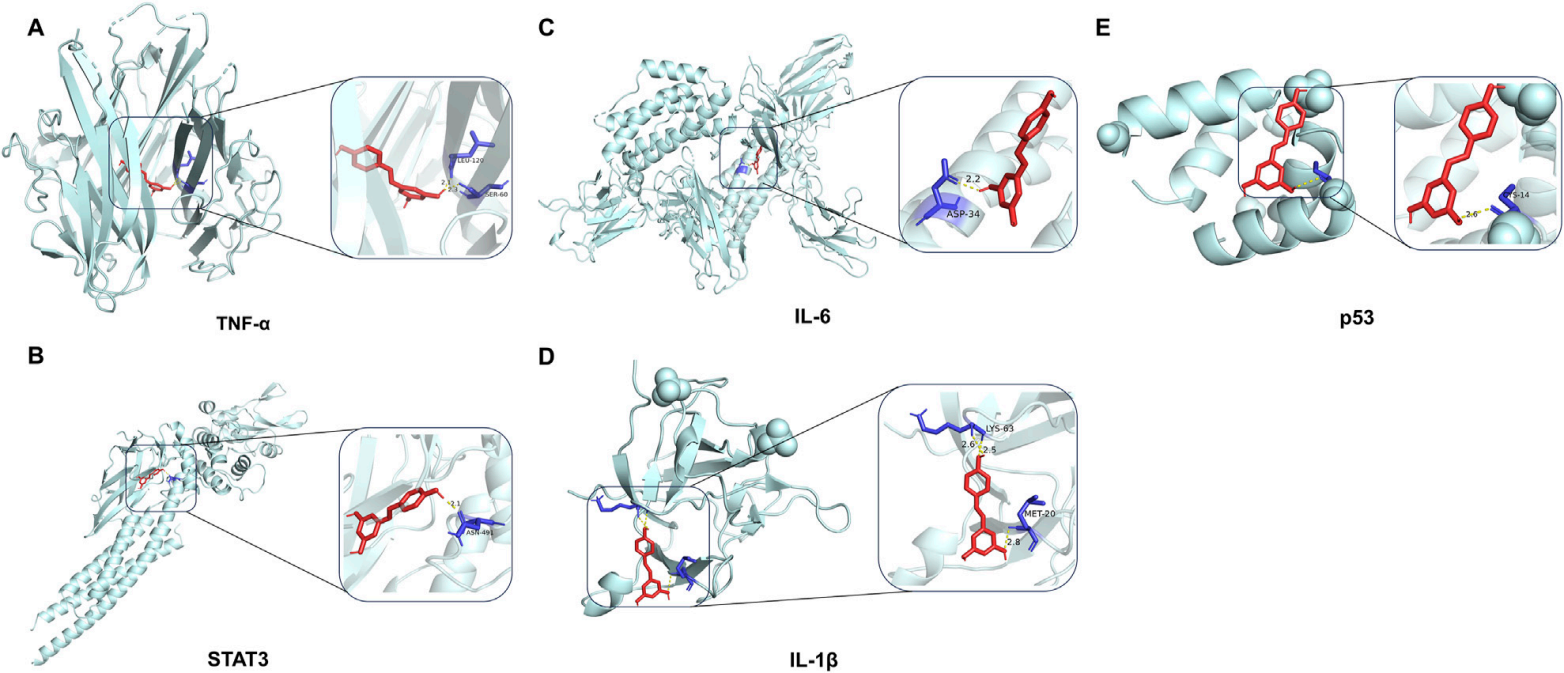
3D map of the binding sites between Res and the top five target proteins: **(A)** TNF-α, **(B)** STAT3, **(C)** IL-6, **(D)** IL-1β, and **(E)** p53. Res is shown in red. Targets are shown in cyan. Hydrogen bonds and key residues are labeled in the diagrams.

**FIGURE 6:**
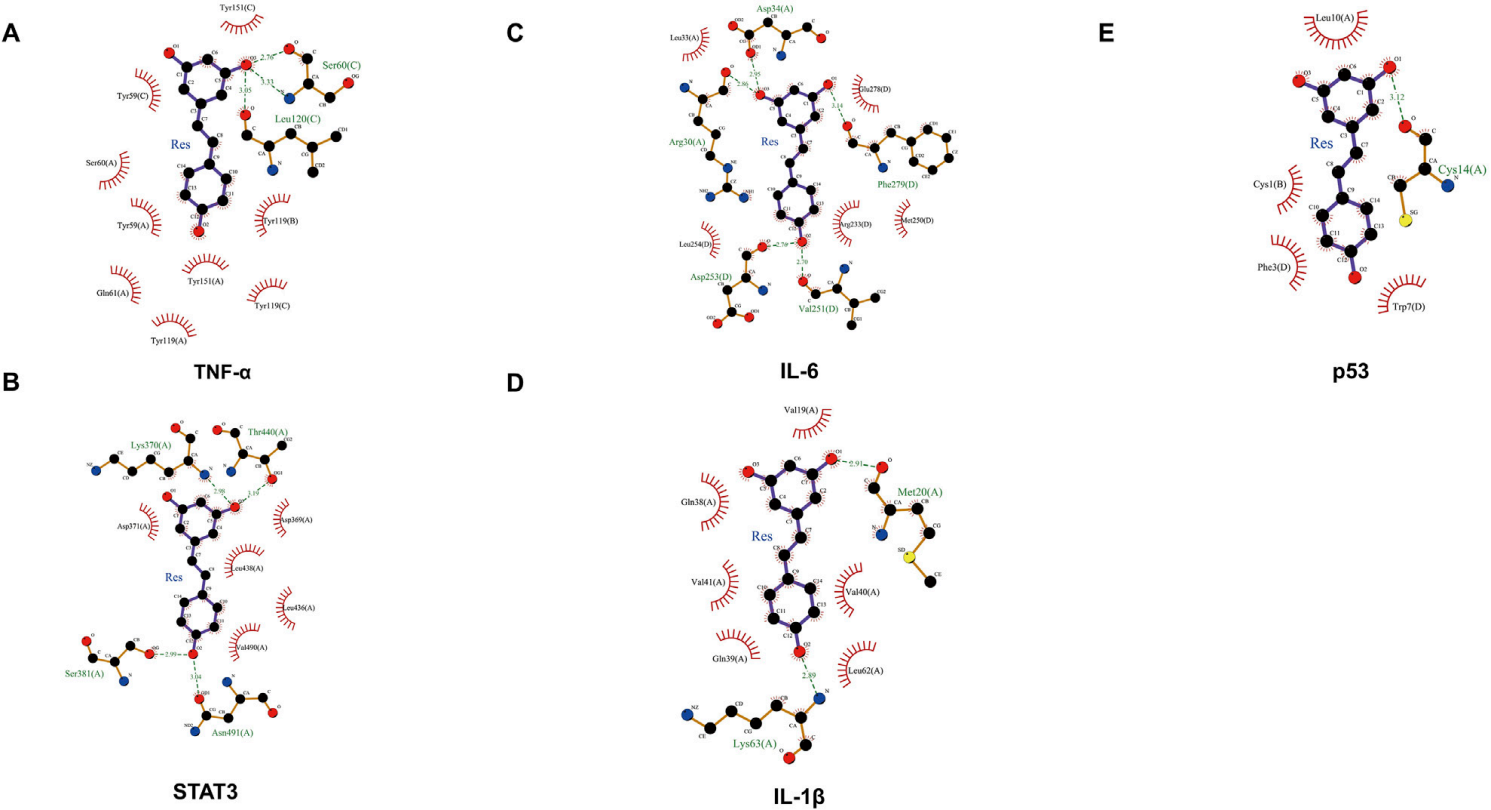
2D map of the binding sites between Res and the top five target proteins: **(A)** TNF-α, **(B)** STAT3, **(C)** IL-6, **(D)** IL-1β, and **(E)** p53.

A molecular docking analysis was conducted on five core target proteins, namely, TNF-α, IL-6, STAT3, p53, and IL-1β. Figure 5 shows the specific docking sites and the number of hydrogen bonds between the Res and the target proteins. Additionally, Figure 6 provides 2D interaction diagrams. In general, the results revealed good binding capabilities between Res and these proteins, with binding energies ≤ −5.8 kcal/mol (Table 1). Notably, the binding affinity of TNF-α was the highest, with a value of −7.9 kcal/mol, where Res stabilized the interface through 2.3 Å (SER-60) and 2.1 Å (LEU-120) hydrogen bonds. In summary, Res demonstrates robust binding affinities with all five core targets.

**TABLE 1:** Detailed presentation of molecular docking results.

Targets	UniProtKB entry	PDB ID	Resolution	Affinity (kcal/mol)
TNF	P01375	7KP9	2.15 Å	−8.9
STAT3	P40763	6NJS	2.7 Å	−6.8
IL6	P05231	5FUC	2.7 Å	−6.5
IL1B	P01584	5R7W	1.27 Å	−6.2
TP53	P04637	6SHZ	1.2 Å	−5.8

### 3.5 Effects of Res on AHR, airway inflammation in mice

**FIGURE 7:**
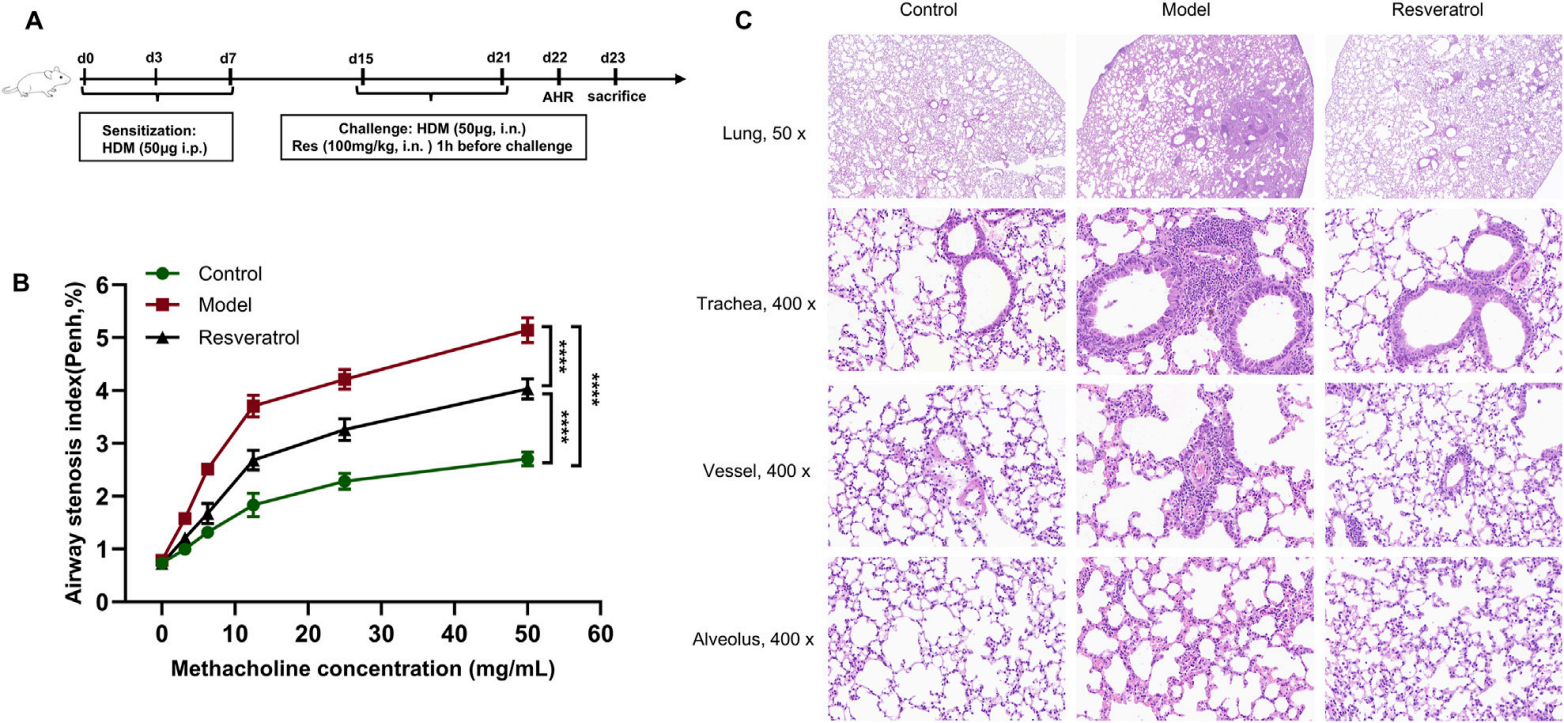
Effects of Res on AHR, lung histopathology. **(A)** Diagram of the experimental protocol. **(B)** Determination of AHR after treatment with methacholine (n = 3). **(C)** H&E staining to determine inflammatory cell infiltration in the lung (×50, 50 magnification; ×400, 400 magnification).

The HDM-induced asthma model was successfully established (Figure 7A). At the baseline, the variation in AHR among the three groups did not attain statistical significance (*p* > 0.05). As the concentration of airway nebulized methacholine increased, AHR increased in all three groups of mice. The overall trend showed that the most obvious elevation of AHR was observed in the model group (Figure 7B). When the concentration of methacholine reached 50 mg/mL, the model group displayed a significantly elevated AHR relative to the control group (*p* < 0.0001), whereas Res intervention induced a significant decline in AHR (*p* < 0.0001). This indicated that AHR was significantly alleviated after Res intervention.

The model group showed significant inflammatory cell infiltration in the peribronchial regions, perivascular areas, and lung parenchyma compared to the control group (Figure 7C). Notably, the inflammatory infiltration was more pronounced in peribronchial tissues than in perivascular compartments. After Res intervention, the inflammatory infiltration around the peribronchial regions, perivascular areas, and lung parenchyma showed significant improvement.

Overall, the control group demonstrated substantial airway inflammatory infiltration and heightened AHR, validating the successful induction of the asthma phenotype in the experimental model. Importantly, the Res group revealed that Res effectively attenuates both airway inflammation and AHR.

### 3.6 Effect of Res on cell viability

**FIGURE 8:**
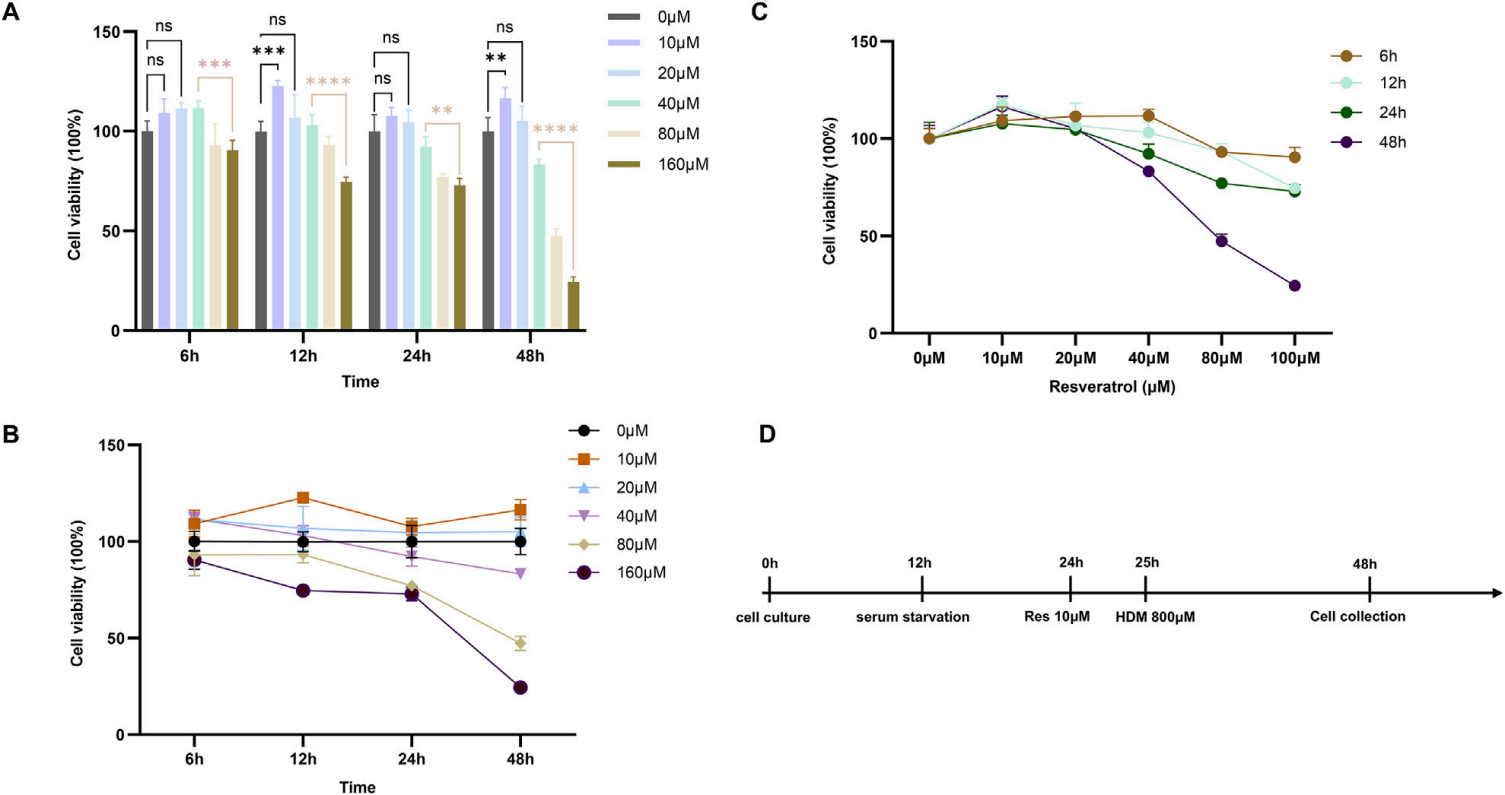
Effect of Res on proliferation of BESE-2B cells. **(A–C)** Changes in cell viability at different concentrations of resveratrol with various interventions, ns: p > 0.05, *: p < 0.05, **: p < 0.01, ***: p < 0.001, and ****: p < 0.0001. **(D)** Diagram of the HDM-induced cell model.

*In vitro*, we systematically assessed the dose- and time-dependent effects of Res on the viability of AECs using CCK-8 assays. Compared to the control group (0 μM), Res showed an effect of promoting cell proliferation within 48 h at concentrations below 20 μM (Figure 8). However, when the concentration was between 40 μM and 160 μM, Res inhibited cell proliferation to a different extent, which was related to the concentration gradient of Res. This is an indication that there is a significant pharmacological toxicity effect on AECs when the Res concentration is greater than 40 μM. In this study, 10 μM Res was chosen as the experimental concentration because it had the most significant effect on promoting cell proliferation at all time points.

### 3.7 Effect of Res on target protein expression *in vivo* and *in vitro*

**FIGURE 9:**
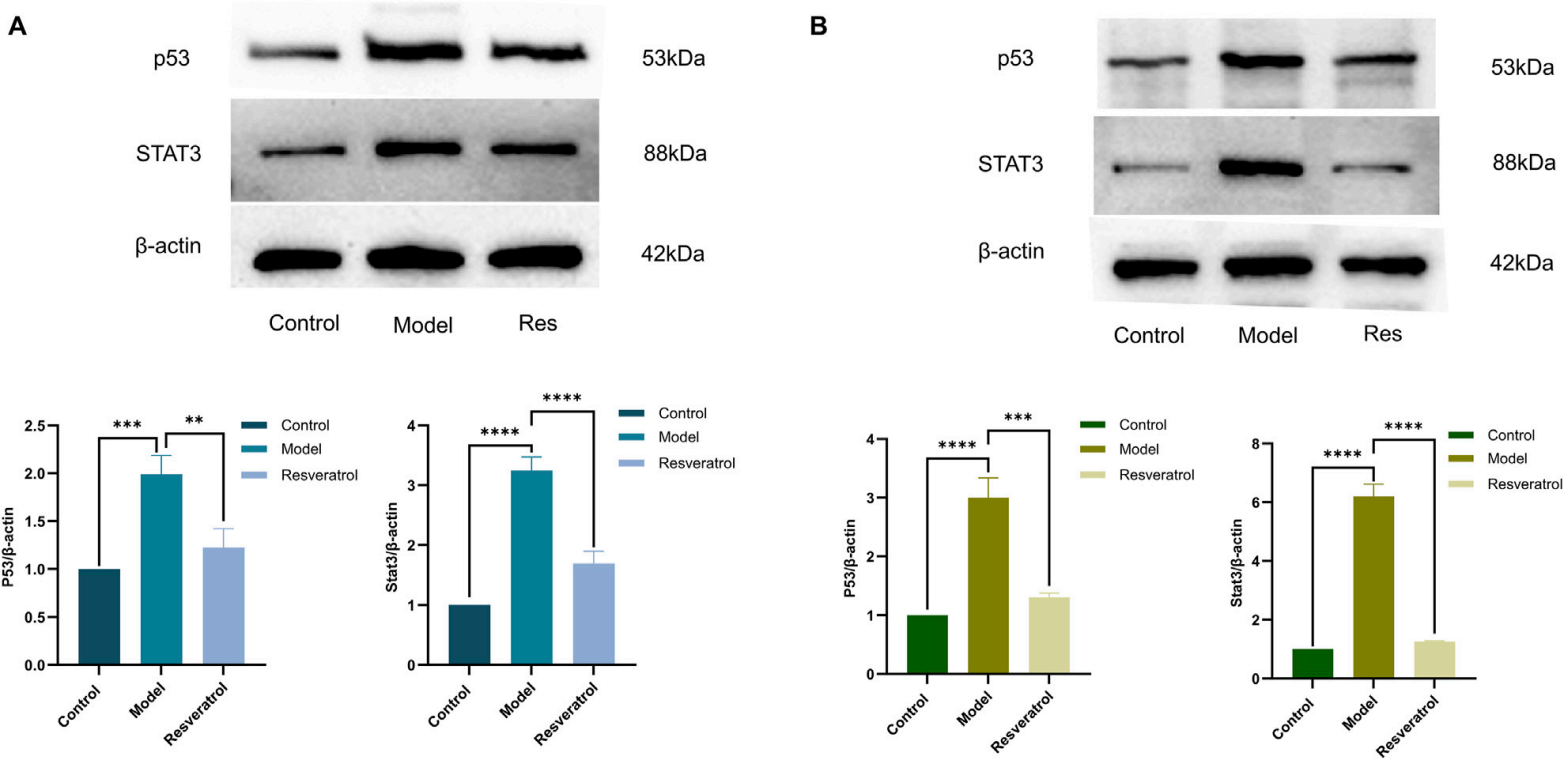
Effect of Res on protein expression of the targets (p53 and STAT3) measured by Western blotting. **(A)** p53 and STAT3 levels in cell supernatants. **(B)** Effect of Res on p53 and STAT3 levels in lung tissue homogenates.

**FIGURE 10:**
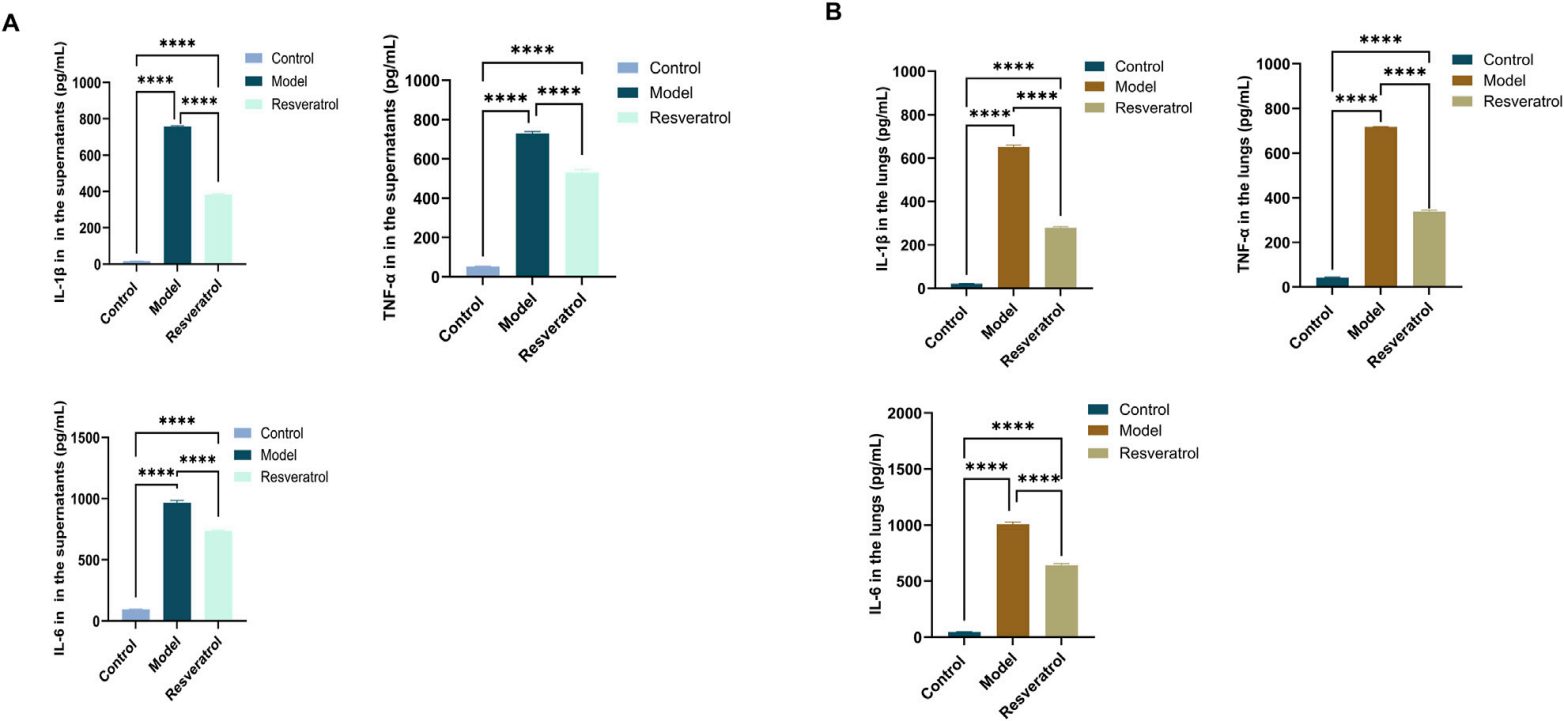
Effect of Res on protein expression of the targets (IL-1β, TNF-α, and IL-6) measured by ELISA. **(A)** IL-1β, TNF-α, and IL-6 levels in cell supernatants. **(B)** IL-1β, TNF-α, and IL-6 levels in lung tissue homogenates.

*In vitro*, treatment of cells with 10 μM Res reversed the abnormal expression of STAT3 and p53 in BEAS-2B cells induced by HDM (Figure 9A). Notably, the expression levels of IL-6, IL-1β, and TNF-α in supernatants, as inflammation factors and target proteins, were also higher when induced by HDM, whereas the expression levels of those proteins decreased with Res intervention (Figure 10A). The results demonstrated that Res downregulated the expression of STAT3, p53, IL-6, IL-1β, and TNF-α, exerting protective effects against HDM-induced AEC injury by suppressing inflammatory responses.

In the lung tissues, STAT3 and p53 protein levels were markedly elevated in the model group compared to the control group. However, administration of Res (100 mg/kg) effectively normalized the overexpression of both STAT3 and p53 (Figure 9B). The concentrations of IL-6, IL-1β, and TNF-α in lung tissue homogenates were higher when induced by HDM, whereas Res decreased the expression levels of those proteins (Figure 10B). In conclusion, Res normalized the expression of the top five targets (TNF-α, IL-6, STAT3, p53, and IL-1β) and alleviated HDM-induced pulmonary inflammation, with consistent inhibitory trends observed in AECs.

## 4 Discussion

The antioxidant, immunomodulatory, and anti-inflammatory properties of dietary polyphenols in chronic inflammatory diseases have been comprehensively investigated (Maleki et al., 2019; Lalit Mohan Nainwal and Arora, 2022; Poonam Arora, 2024). Of note, Res, a representative small polyphenolic molecule within this class, exhibits a promising therapeutic potential for the treatment of respiratory diseases (Yang et al., 2010; Wang et al., 2017; Wang et al., 2018). Notably, Res has been demonstrated in multiple studies to alleviate airway inflammation and AHR in asthma (Lee et al., 2009; Royce et al., 2011; Chen et al., 2015; Lee et al., 2017; Jiang et al., 2019). Nevertheless, the mechanisms underlying these therapeutic effects remain incompletely described, hampering the translational development of Res into clinical practice.

In this study, network pharmacological analysis highlighted TNF, IL6, STAT3, TP53, and IL1B as the top five core targets of Res, suggesting their potential role in asthma treatment. Further exploration of the specific binding sites for Res was performed using molecular docking validation, which indicated that the top five core targets exhibited strong binding affinity for Res. Finally, the relevant targets were validated using both HDM-induced mouse models and AEC models.

HDM is one of the most common sources of indoor allergens and is closely associated with the development of asthma (Calderón et al., 2015). In comparison with ovalbumin (OVA), HDM has been shown to induce a more clinically relevant animal model of asthma. The current study revealed marked airway inflammatory infiltration and elevated AHR in HDM-stimulated mice, aligning with prior findings (Hammad et al., 2009; Chen et al., 2015; Shim et al., 2022). Notably, Res significantly attenuated airway inflammation and AHR *in vivo*. Structural and functional impairment of AECs initiates asthma pathogenesis by driving cytokine-mediated activation of innate and adaptive immune pathways, triggering inflammatory cascades (Yang et al., 2021; Qin et al., 2024). *In vitro*, our data demonstrated that Res enhanced AEC viability in a concentration-dependent manner, peaking at 10 μM. Furthermore, Res reversed HDM-induced aberrant protein expression of TNF-α, IL-6, STAT3, p53, and IL-1β both *in vivo* and *in vitro*.

STAT3 single-nucleotide polymorphism is associated with decreased lung function in asthma patients (Litonjua et al., 2005). The activation of Th2, Th17 cells, and macrophages is mediated by STAT3, which also drives the pulmonary infiltration of neutrophils and eosinophils (Chaudhry et al., 2009; Stritesky et al., 2011; Nakamura et al., 2015; Wei et al., 2018). Studies have shown that Res attenuates inflammatory responses by modulating STAT3 expression. Res may prevent acute lung injury induced by zinc chloride fumes by downregulating STAT3 (Wang et al., 2022). Another study showed that Res downregulated STAT3 expression and attenuated the inflammatory reaction observed in RAW264.7 macrophages stimulated with lipopolysaccharide (Ma et al., 2017). However, limited studies have investigated the function of STAT3 in Res-mediated asthma treatment. Our study demonstrates that Res reduces the expression of STAT3 proteins in lung tissue and AECs. In addition, the JAK–STAT signaling pathway was identified as one of the enriched pathways. Collectively, the downregulation of STAT3 expression and its associated signaling pathways likely underlie Res’s therapeutic effects in asthma treatment.

TP53 governs multiple biological processes, including the induction of cell-cycle arrest, senescence, and apoptosis (Li et al., 2012). In addition, TP53 participates in immune regulation, inflammation generation, and other related processes (Chamoto et al., 2020). Res can significantly induce cell-cycle arrest and apoptosis in eosinophils from asthma patients by increasing the levels of p53 and p21 (Hu et al., 2016). Our data demonstrated a significant reduction in p53 protein expression by Res in lung tissue and AECs. Moreover, our findings showed that KEGG enrichment analysis identified the apoptosis and p53 signaling pathways. These findings suggest that Res may attenuate asthmatic airway inflammation by modulating p53 expression and related pathways.

As secreted proteins, IL-6, TNF-α, and IL-1β, detected by ELISA, serve as indicators of both the regulatory effects of Res on target gene expression and inflammatory responses. IL-1β is an important regulator of AHR and Th2 cell-associated inflammatory cytokine production (Schmitz et al., 2003; Liao et al., 2015; Burbank et al., 2021). Res attenuates IL-1β expression in AECs induced by cigarette smoke extract (Zeng et al., 2022). This study provides the first evidence that Res attenuates IL-1β expression in asthmatic mice, as shown by reduced IL-1β levels in the lung tissue and cell supernatants. IL-6 modulates CD4^+^ Th2 and Th17 cell activity, directly influencing T-cell differentiation processes (Jones et al., 2010; Smith and Maizels, 2014; Gubernatorova et al., 2018). IL-6 was elevated in asthmatics and correlated with disease severity (Virchow et al., 1996; Morjaria et al., 2011). In a PM2.5-induced guinea pig model of asthma, Res reduced the expression level of IL-6 in bronchoalveolar lavage fluid (Morales-Rubio et al., 2022). Another study demonstrated that Res significantly reduced IL-33-induced IL-6 production, which is linked to mast cell-mediated inflammation (Xu et al., 2020). The present study showed that Res reduced IL-6 expression in lung tissue and cell supernatants. As a key pro-inflammatory cytokine, TNF-α stimulates the secretion of various inflammatory molecules, thereby exacerbating inflammatory cascades (Lykouras et al., 2008; Malaviya et al., 2017; Gough and Myles, 2020; Alshevskaya et al., 2022; Arora et al., 2022b; Arora et al., 2022c). Res was found to reduce the level of TNF-α expression in the lungs (Chen et al., 2015; André et al., 2016; Jiang et al., 2019; Morales-Rubio et al., 2022). Furthermore, Res has been demonstrated to attenuate the expression levels of TNF-α in AECs induced by cigarette smoke extract (Zeng et al., 2022). Consistent with previous studies, the present study showed that Res reduced TNF-α expression in lung tissue and cell supernatants.

Pathway analysis revealed that TNF-α, IL-6, and IL-1β—the core targets identified in our study—are integral components of the TNF signaling pathway. The TNFR1 signaling axis primarily orchestrates the stimulation of NF-κB and MAPK cascades (Sabio and Davis, 2014; Varfolomeev and Vucic, 2018). Res has been reported to suppress both NF-κB and MAPK pathways. An experimental study showed that Res ameliorated airway inflammation and structural remodeling *via* targeting the HMGB1/TLR4/NF-κB pathway (Jiang et al., 2019). Another study demonstrated that Res suppressed the secretion of Th2 cytokines from RBL-2H3 cells *via* the MAPK signaling pathway (Han et al., 2015). Our findings extend these observations by demonstrating Res’s ability to downregulate TNF-α, IL-6, and IL-1β in mice and AECs. Whereas direct inhibition of TNFR1 signaling intermediates was not assessed here, the coordinated reduction of these downstream cytokines strongly suggests Res’s multitiered modulation of the TNF pathway. This aligns with its pleiotropic anti-inflammatory effects.

As far as we are aware, previous studies have not systematically explored both the identification and functional validation of Res’s therapeutic targets in asthma. The strength of this study is that we comprehensively identified the core targets of Res for asthma using computational and experimental methods.

Nevertheless, certain limitations exist in this study. First, following the identification of target genes, the associated signaling pathways were not experimentally validated in this study. Second, a comparative analysis between Res and other medications employed in the treatment of asthma was not conducted. Furthermore, a combined intervention was not implemented to provide a more comprehensive evaluation of the effect of Res on asthma.

Therefore, further validation of the pathways is essential, and the mechanisms and effects of Res in asthma treatment should be studied in combination with conventional asthma medications. Finally, there is a paucity of relevant clinical trials, and the optimal dose and bioavailability of Res still need to be determined by animal and clinical experiments.

## 5 Conclusion

In this paper, we comprehensively identified Res targets for asthma using network pharmacology combined with experimental validation. Res treatment reversed the aberrant protein expression of genes such as TNF, IL6, STAT3, TP53, and IL1B. It was also further confirmed that Res could reduce asthma airway inflammation and AHR. The present study comprehensively revealed the potential targets of Res in asthma and provided a solid foundation for further application of Res in clinical practice.

## Data Availability

The original contributions presented in the study are included in the article/Supplementary Material; further inquiries can be directed to the corresponding author.
