# Supplementary figures and images for: Uncovering the mechanism of resveratrol in the treatment of asthma: a network pharmacology approach with molecular docking and experimental validation
Source: Front Pharmacol. 2025 May 16;16:1596737. doi: 10.3389/fphar.2025.1596737 (PMC12122464; doi:10.3389/fphar.2025.1596737)

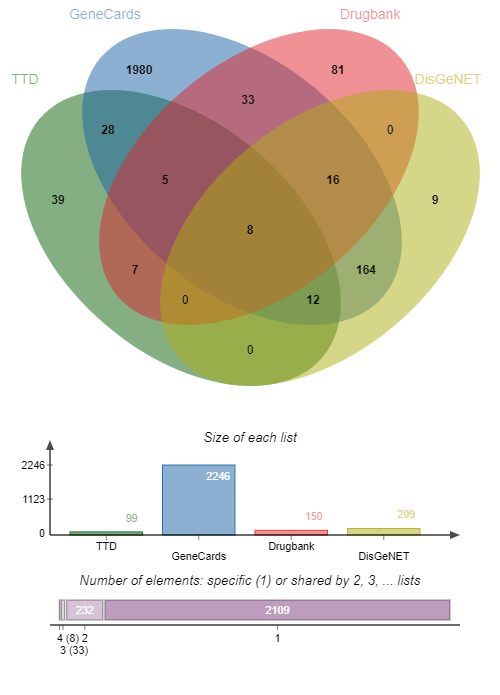

Supplement: Supplementary file 1 [file DataSheet1.zip › Supplementary data/network pharmacology data/asthma targets/targets of asthma.png]

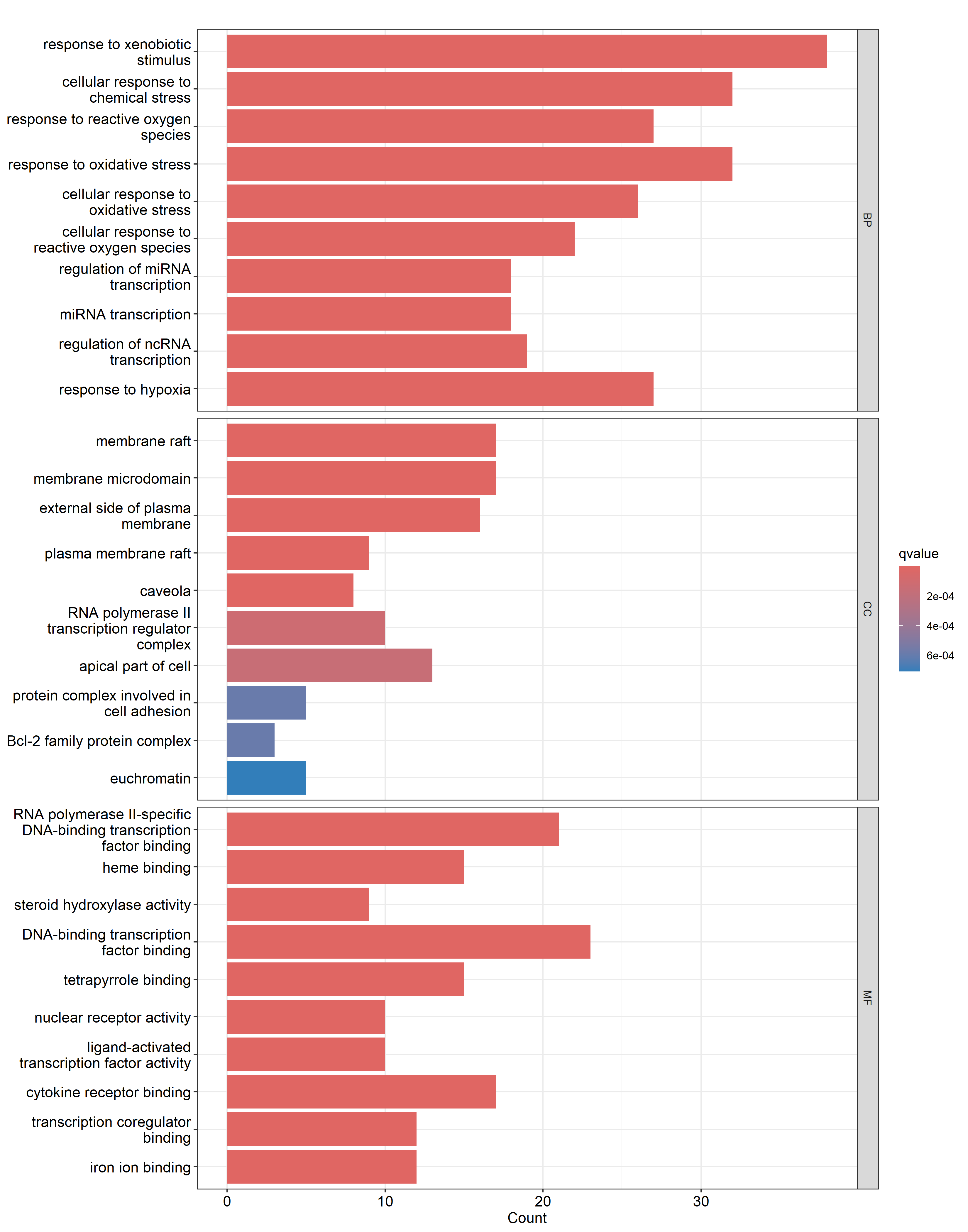

Supplement: Supplementary file 1 [file DataSheet1.zip › Supplementary data/network pharmacology data/GO results/barplot.tiff]

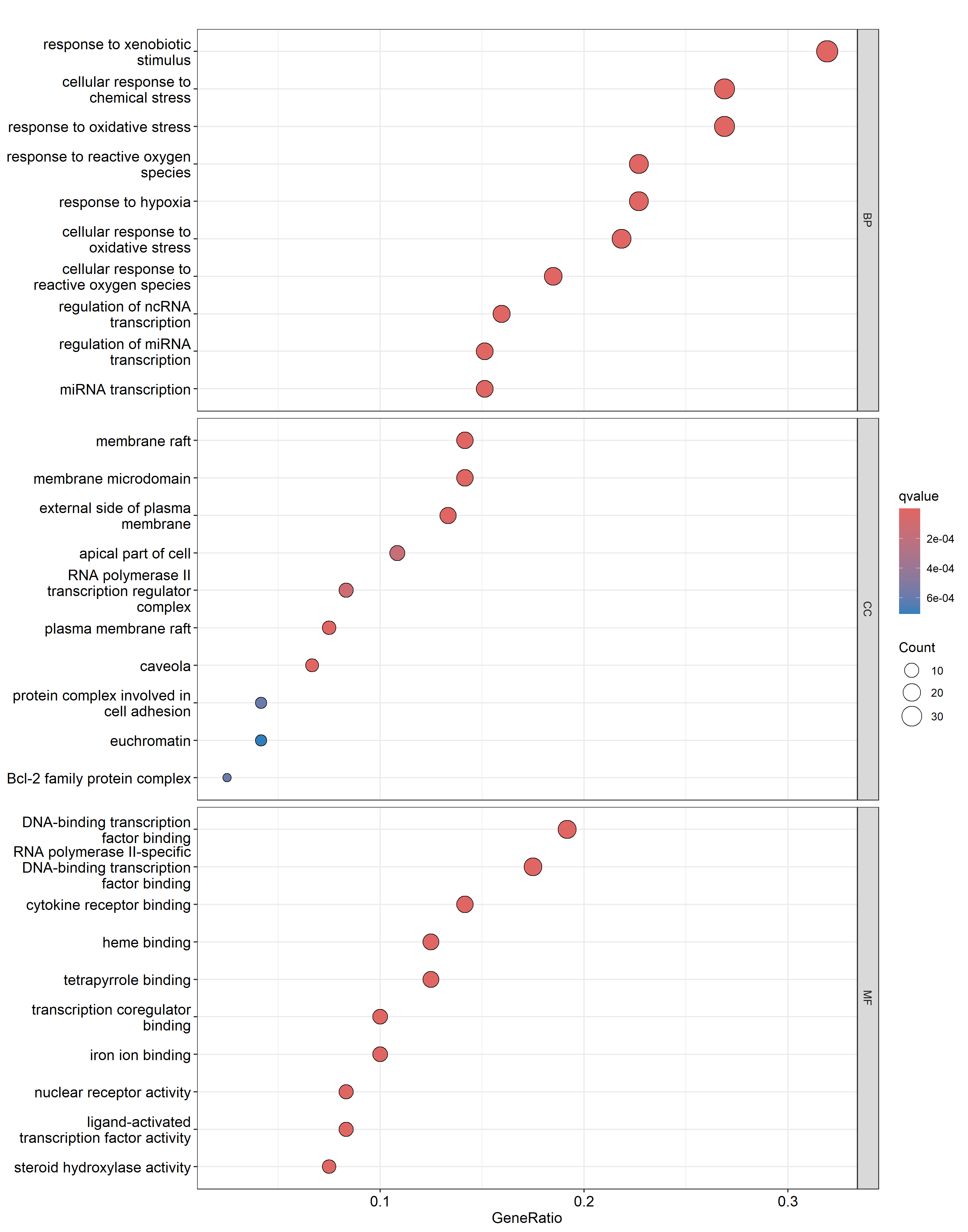

Supplement: Supplementary file 1 [file DataSheet1.zip › Supplementary data/network pharmacology data/GO results/bubble.tiff]

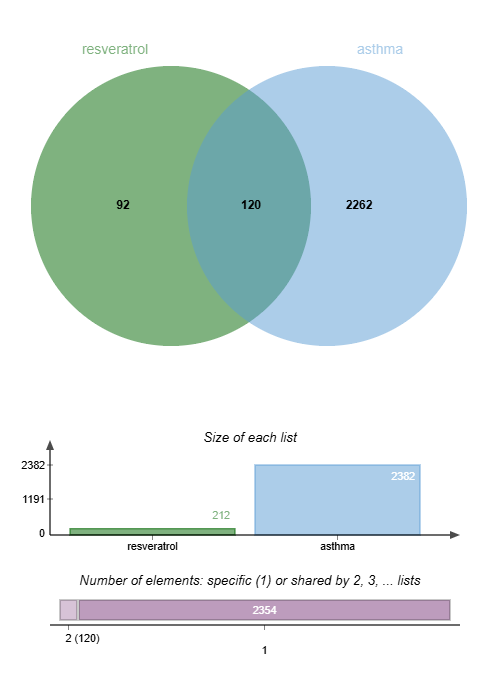

Supplement: Supplementary file 1 [file DataSheet1.zip › Supplementary data/network pharmacology data/Intersection gene/Intersection gene.png]

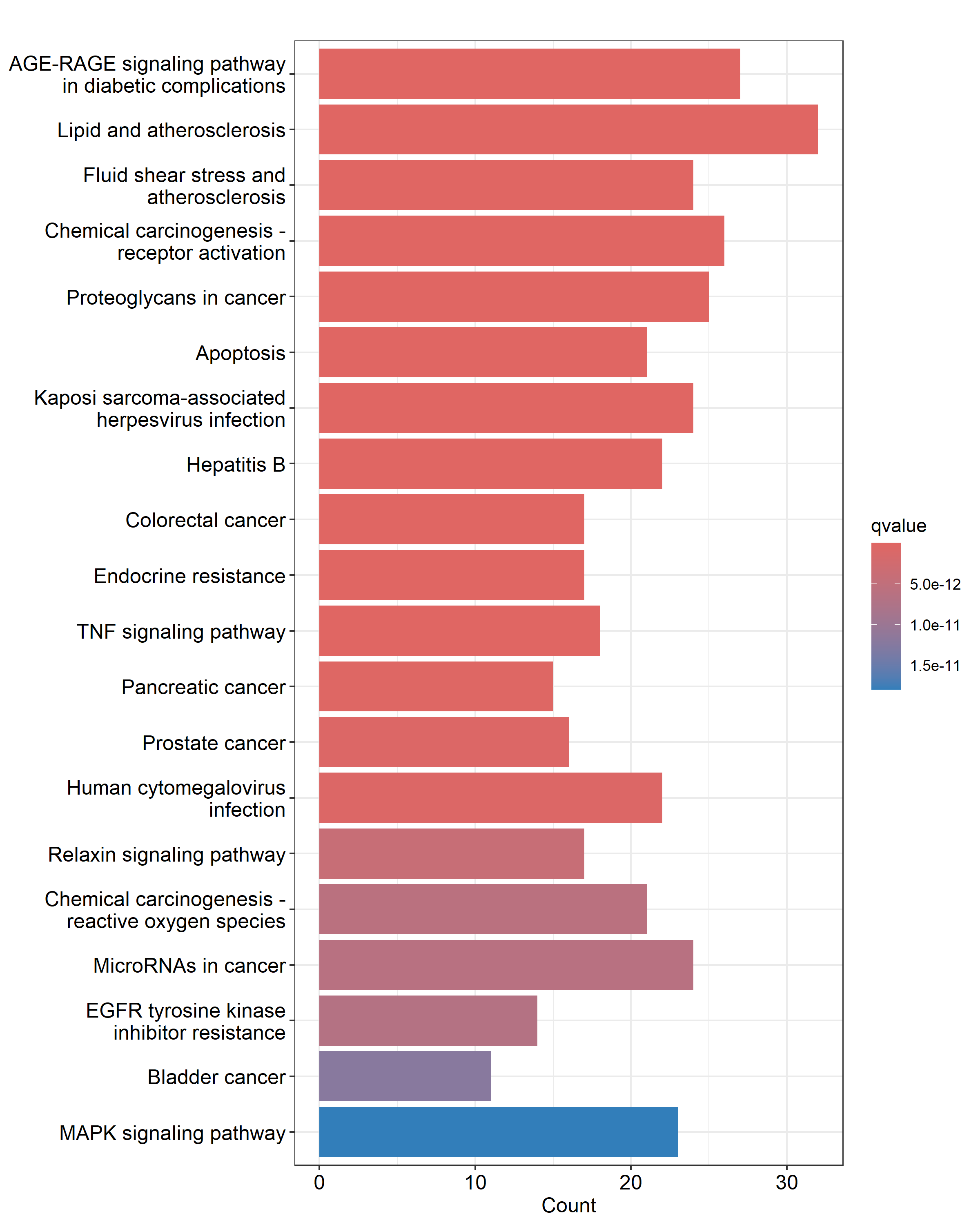

Supplement: Supplementary file 1 [file DataSheet1.zip › Supplementary data/network pharmacology data/KEGG resullts/barplot.tiff]

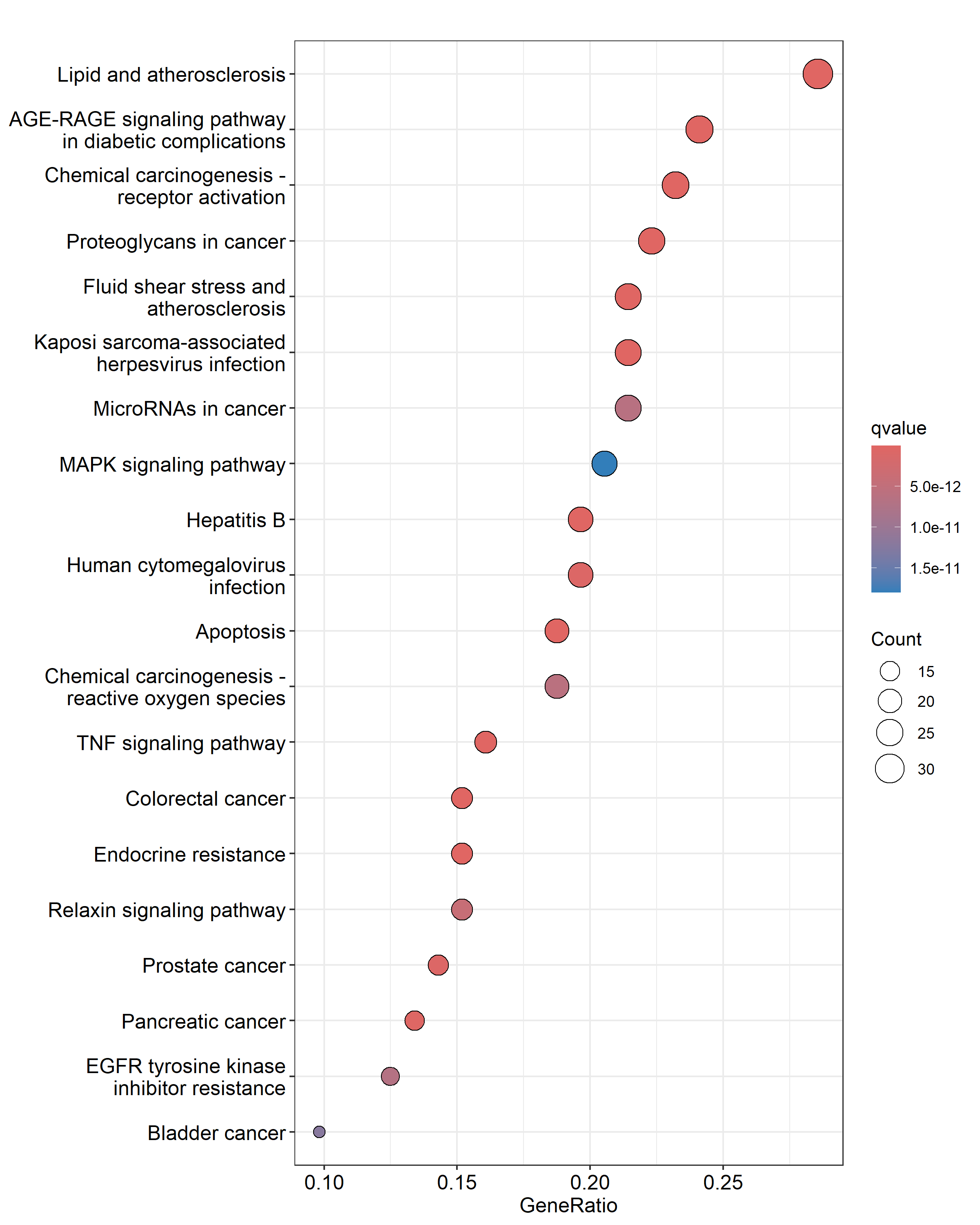

Supplement: Supplementary file 1 [file DataSheet1.zip › Supplementary data/network pharmacology data/KEGG resullts/bubble.tiff]

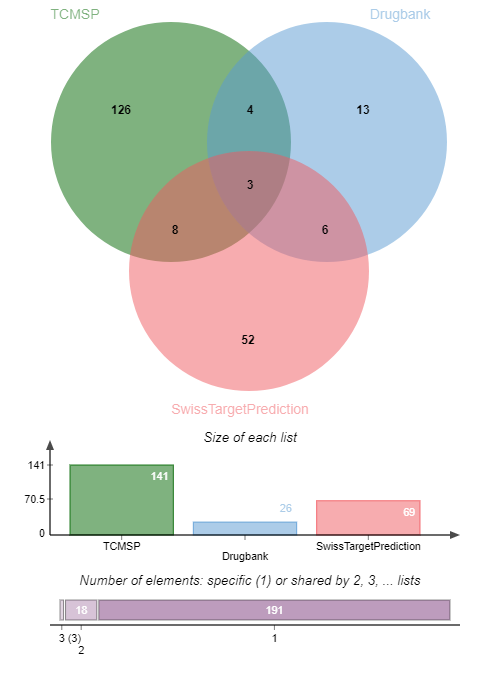

Supplement: Supplementary file 1 [file DataSheet1.zip › Supplementary data/network pharmacology data/Res targets/venn-Res.png]

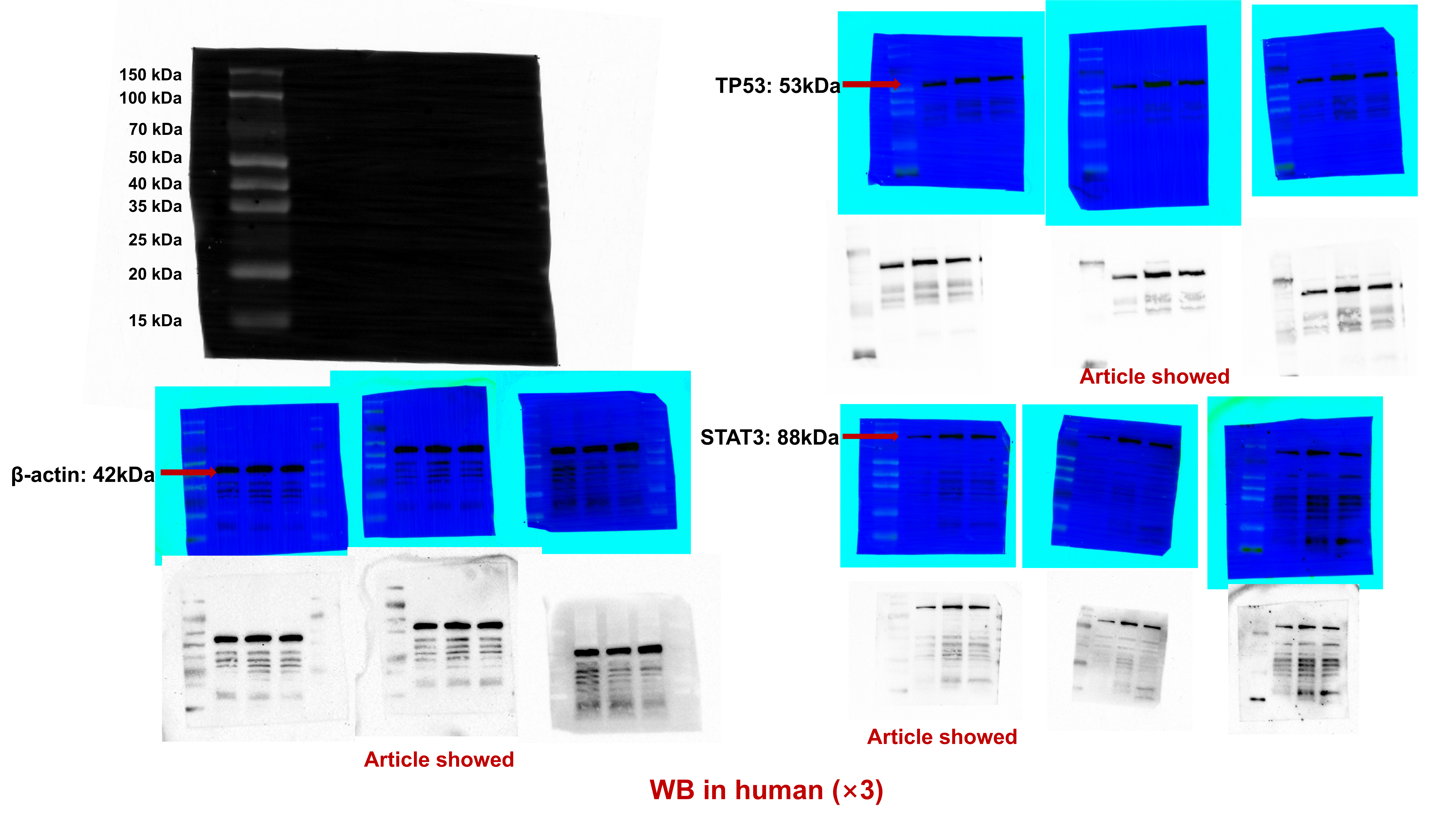

Supplement: Supplementary file 1 [file DataSheet1.zip › Supplementary data/WB data/WB in human (×3).tif]

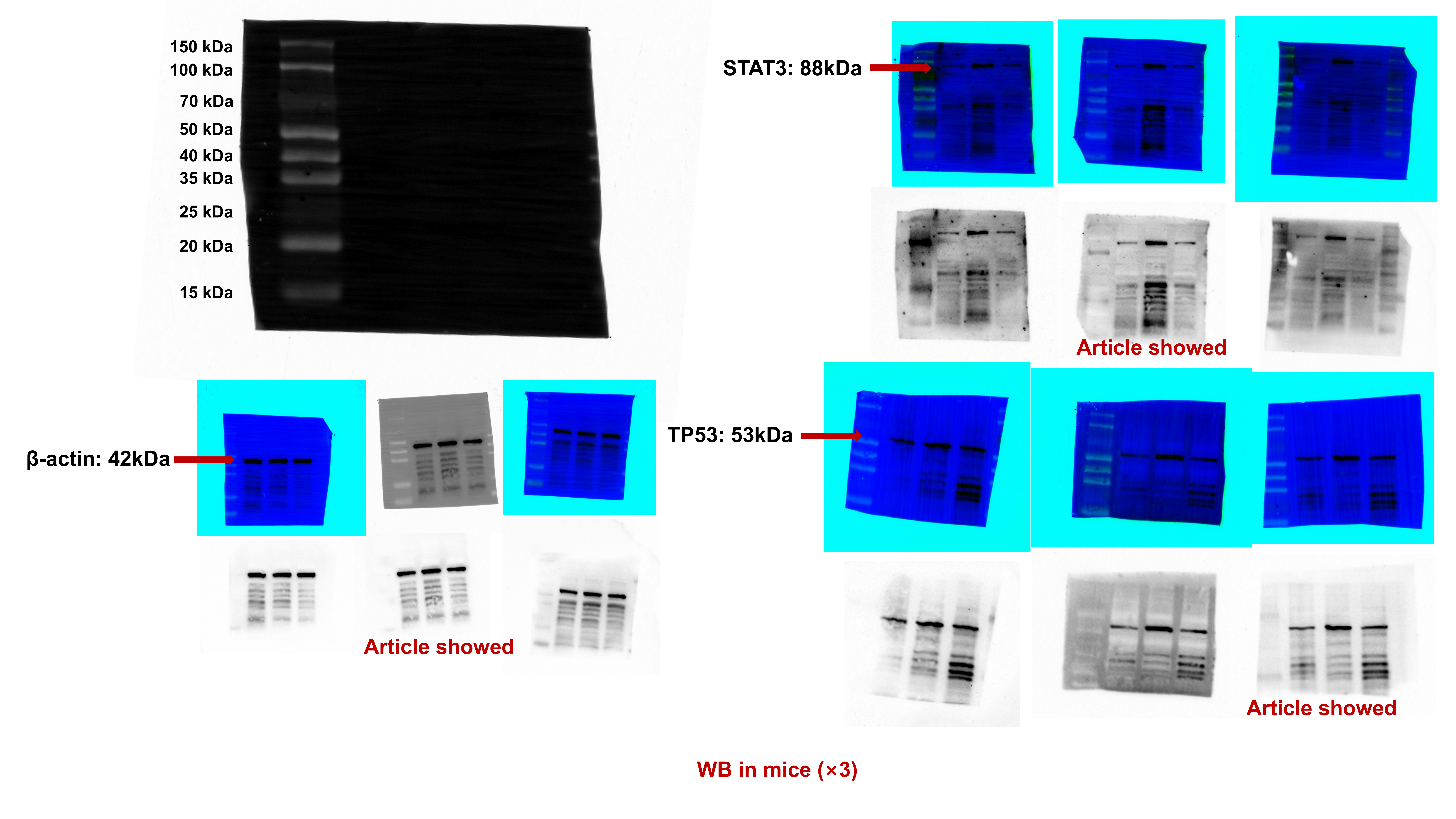

Supplement: Supplementary file 1 [file DataSheet1.zip › Supplementary data/WB data/WB in mice (×3).tif]
